# Screening HCC Patients Who Can Benefit from the Sequential Treatment of Conversion Therapy and Radical Surgery and Constructing a Predictive Model

**DOI:** 10.3390/cancers17243928

**Published:** 2025-12-08

**Authors:** Lei Liu, Chuan Qin, Kai Lei, Han Zhang, Wenqian Zhang

**Affiliations:** 1 Department of Hepatobiliary Surgery, Chongqing University Three Gorges Hospital, Chongqing 404031, China; 2 Department of Gastrointestinal Surgery, Chongqing University Three Gorges Hospital, Chongqing 404031, China; 3 Department of Hepatobiliary Surgery, The Second Affiliated Hospital of Chongqing Medical University, Chongqing 400000, China; 4 Department of Oncology, Chongqing University Three Gorges Hospital, Chongqing 404031, China; 5 Department of Nuclear Medicine, The Second Affiliated Hospital of Chongqing Medical University, Chongqing 400000, China

**Keywords:** conversion therapy, hepatocellular carcinoma (HCC), nomogram, radical surgical resection, transcatheter arterial chemoembolization (TACE)

## Abstract

Background/Objective: This study aimed to develop a new nomogram to predict the likelihood of benefit for patients with advanced HCC who can benefit from the sequential treatment of conversion therapy and radical surgery. Materials and Methods: A total of 589 patients who met the criteria were included in this study. According to a 7:3 ratio, the development group included 412 patients, while the validation group included 177 patients. The clinical outcome set for this study was whether one could benefit from radical resection after conversion therapy. Independent risk factors for the outcome were screened through univariable and multivariable Logistic regression analysis. We constructed a nomogram using independent risk factors. The areas under the receiver operating characteristic (ROC) curve, calibration curve, and clinical decision curve were used to evaluate the performance of the model. Results: Sex (OR = 2.000; 95% CI: 1.018, 3.931; *p* = 0.044), AFP (OR = 0.533; 95% CI: 0.313, 0.907; *p* = 0.020), targeted therapy and immunotherapy (OR = 3.283; 95% CI: 2.029, 5.312; *p* < 0.001), and postoperative TACE (OR = 6.544; 95% CI: 4.021, 10.649; *p* < 0.001) were identified as independent risk factors for determining whether there was a benefit. The AUCs of the nomogram for the development set and the validation set were 0.793(95% CI: 0.749–0.836) and 0.770 (95% CI: 0.697–0.844), respectively. The calibration curves and clinical decision curves of the nomogram indicate that it has good predictive performance. Conclusions: This study developed a nomogram for predicting whether HCC patients can benefit from the sequential treatment of conversion therapy and radical surgery. This is of great help for us to formulate treatment strategies for patients with advanced HCC.

## Simple Summary

The prognosis of hepatocellular carcinoma is poor. Radical surgery after conversion therapy may potentially improve the prognosis of patients with hepatocellular carcinoma. Therefore, this study aimed to develop a new nomogram to predict the possibility of benefit for patients with advanced HCC in the sequential treatment of conversion therapy and radical surgery. Through the evaluation of the area under the ROC curve, calibration curve, and decision curve analysis, the nomogram constructed in this study has good predictive ability. This is of great help for us to formulate treatment strategies for patients with advanced HCC.

## 1. Introduction

Hepatocellular carcinoma (HCC) is one of the leading causes of cancer-related deaths worldwide [1]. The burden of HCC is closely associated with the prevalence of hepatitis B virus (HBV), alcoholic liver disease, and metabolic Dysfunction-Associated Steatotic Liver Disease (MASLD). HCC remains a significant global health burden. As one of the most common malignant tumors worldwide, HCC often presents with a latent onset, and most patients are diagnosed at an advanced stage, losing the opportunity to receive radical treatments (such as surgical resection, liver transplantation, or radiofrequency ablation) [2,3,4]. For these patients, transcatheter arterial chemoembolization (TACE) has become the standard non-radical treatment option for advanced HCC [5,6]. Moreover, numerous studies have demonstrated that TACE, as a local treatment method, can significantly improve the prognosis of patients with advanced HCC. With the continuous evolution of liver cancer treatment concepts, the role of TACE has no longer been limited to palliative treatment but has instead emerged as a crucial transformational treatment strategy, showing great potential. Therefore, an increasing number of studies have begun to focus on TACE as a transformative treatment that offers a chance for radical treatment for patients with advanced HCC [7,8,9].

In response to this, this study identified patients with HCC who can benefit from the sequential treatment of conversion therapy and radical surgery. Based on clinical characteristics, a nomogram was constructed to predict the probability that patients with advanced-stage HCC would benefit from the sequential treatment of conversion therapy and radical surgery.

## 2. Materials and Methods

### 2.1. Patient Selection

This study included patients from the Second Affiliated Hospital of Chongqing Medical University who met the inclusion and exclusion criteria during the period from 2010 to 2022. The inclusion criteria for this study were as follows: (1) age between 18 and 80 years old; (2) both a clinical diagnosis and a postoperative pathological diagnosis of HCC; (3) radical resection surgery having been undergone; (4) receipt of TACE conversion therapy before radical resection surgery; (5) a Child–Pugh score for liver function of A or B; (6) receipt of regular follow-up; (7) complete clinical baseline data. The exclusion criteria for this study were as follows: (1) receipt of liver transplantation or radiofrequency ablation for liver lesion treatment before undergoing liver resection surgery; (2) re-resection of the liver after recurrence following the initial liver resection surgery; (3) a Child–Pugh score for liver function of C; (4) a history of malignant tumors in other organs and systems; (5) a history of serious underlying diseases; (6) death during the perioperative period.

All patients who met the inclusion criteria and did not meet the exclusion criteria were randomly divided into a development set and a validation set in a 7:3 ratio.

### 2.2. Data Collection and Follow-Up

The baseline patient data that we needed to collect included sex, age, Barcelona Clinic Liver Cancer (BCLC) stage, Child–Pugh score, tumor number, max. tumor size, alpha-fetoprotein (AFP) level, alanine aminotransferase (ALT) level, aspartate transferase (AST) level, total bilirubin (TBIL) level, prognostic nutritional index (PNI), preoperative TACE number, tumor differentiation, microvascular invasion (MVI), targeted therapy and immunotherapy, postoperative TACE, survival status, and overall survival (OS).

The clinical outcome set for this study was whether one could benefit from the sequential treatment of conversion therapy and radical surgery. The conversion therapy in this study was a simple TACE therapy. After the conversion therapy, an assessment of the tumor treatment was conducted. The treatment effect was evaluated one month after TACE treatment. Contrast-enhanced computed tomography (CT) or magnetic resonance imaging (MRI) was used to assess the liver tumor lesions of the patients. The assessment criteria referred to mRECIST. Patients who met the criterion of Partial Response (PR) or above were included in the radical surgery. A routine inpatient follow-up visit was conducted one month after the radical surgery. The TACE performed after surgery included in this study was for adjuvant treatment, not for recurrent treatment. Adjuvant therapy was provided based on the patient’s pathological condition and high-risk recurrence factors. If the patient had a factor such as a large tumor, multiple tumors, microvascular invasion, poor tumor differentiation, or extremely high preoperative AFP levels, adjuvant TACE treatment was administered after radical surgery.

The criterion for benefit was to achieve an OS of two years after the sequential treatment of conversion therapy and radical surgery. In this study, the median OSs of the patients included in the development set and the validation set were 22.0 and 19.0, respectively, which were close to a two-year survival period. And numerous real-world studies have shown that the median survival time for patients in the BCLC B or C stage is approximately two years. In order to ensure a certain positive outcome for the selection of prognostic variables, the end-point “benefit” time point in this study was set at the two-year survival time. OS is defined as the time interval from the start of radical treatment to death. If there is no death, it is the time interval from the start of radical treatment to the end of the follow-up period. The deadline for the follow-up was August 2025. The follow-up for all patients was conducted through hospital records, telephone calls, or outpatient visits.

TACE operation procedure: The right femoral artery was punctured using the Seldinger technique. A 5 Fr sheath was introduced over a short guidewire, followed by the advancement of a 5 Fr angiography catheter under X-ray fluoroscopy into the abdominal trunk and common hepatic artery for angiographic visualization of the tumor vasculature. A 2.7 Fr microcatheter was then selectively navigated into the tumor-feeding arteries using a micro-guidewire. An emulsion of pirarubicin (20 mg) and iodized oil (5–20 mL) was administered through the microcatheter. Subsequent embolization was performed with microspheres, the size of which was selected based on tumor burden and blood supply. The procedure’s endpoint was confirmed five minutes post-embolization by digital subtraction angiography (DSA), which demonstrated stasis of flow in the target arteries.

In this study, the purpose of the radical surgeries performed on the patients was to completely remove the malignant tumors in the liver through the operations and to ensure that there were no residual cancer cells at the surgical margins. Therefore, due to the different locations of the tumors in each patient, the types of radical surgeries they underwent also varied, including partial liver resection, segmental liver resection, and local liver resection, etc.

### 2.3. Statistical Analysis

Regarding the baseline data of the patients, continuous numerical variables that followed a normal distribution were statistically analyzed using the mean ± standard deviation. Continuous numerical variables that did not follow a normal distribution were statistically analyzed using the median (and interquartile range). Categorical variables were statistically analyzed using counts and percentages. When comparing the baseline data between the development set and the validation set, continuous numerical variables that followed a normal distribution were analyzed using the independent-sample *t*-test, while those that did not follow a normal distribution were analyzed using the Mann–Whitney U test. Categorical variables were analyzed using the chi-square test. 

The screening of independent risk factors was conducted using univariable and multivariable Logistic regression analysis. Variables with a *p* value less than 0.05 in the single-factor analysis were included in the multivariable analysis. The independent risk factors identified through univariable and multivariable Logistic regression analyses were used to construct a nomogram. The nomogram was evaluated and validated through the ROC curves, calibration curves, and clinical decision curves. SPSS 26.0 (IBM, Chicago, IL, USA) and R software 4.3.0 (http://www.r-project.org/, accessed on 1 September 2025) were used for statistical analysis. A *p* value less than 0.05 was considered statistically significant in this study.

## 3. Results

### 3.1. Patient Baseline Data

A total of 589 patients who met the criteria were included in this study. According to a 7:3 ratio, the development group included 412 patients, while the validation group included 177 patients. The baseline data of the patients in the development set and the validation set are presented in Table 1. It can be observed that, apart from the differences in receiving targeted therapy and immunotherapy (*p* = 0.041) as well as survival status (*p* = 0.032) between the development set and the validation set, there are no significant differences in the comparison of the remaining variables. In the development set and the validation set, there were 284 (68.9%) and 123 (69.5%) patients, respectively, in the BCLC B/C stage. In the development set and the validation set, 172 (41.7%) and 62 (35.0%) patients, respectively, were able to receive radical treatment after the conversion therapy and achieved a two-year OS. The median OS for the patients in the development set and the validation set was 22.0 (15.0–36.0) and 19.0 (14.0–32.5) months, respectively.

### 3.2. Screening of Independent Risk Factors

As shown in Table 2, regarding the outcome of whether one could benefit from the sequential treatment of conversion therapy and radical surgery, the variables of sex, age, BCLC stage, Child–Pugh score, tumor number, max. tumor size, AFP, ALT, AST, TBIL, PNI, preoperative TACE number, tumor differentiation, MVI, targeted therapy and immunotherapy, and postoperative TACE were included in the univariable Logistic regression analysis of the development set. Among them, sex (*p* = 0.015), BCLC stage (*p* = 0.001), tumor number (*p* = 0.018), max. tumor size (*p* < 0.001), AFP (*p* = 0.001), ALT (*p* = 0.013), AST (*p* = 0.002), PNI (*p* = 0.032), preoperative TACE number (*p* = 0.035), targeted therapy and immunotherapy (*p* < 0.001), and postoperative TACE (*p* < 0.001) were included in the multivariable Logistic regression analysis. Ultimately, sex (OR = 2.000; 95% CI: 1.018, 3.931; *p* = 0.044), AFP (OR = 0.533; 95% CI: 0.313, 0.907; *p* = 0.020), targeted therapy and immunotherapy (OR = 3.283; 95% CI: 2.029, 5.312; *p* < 0.001), and postoperative TACE (OR = 6.544; 95% CI: 4.021, 10.649; *p* < 0.001) were identified as independent risk factors for the clinical outcome in this study.

### 3.3. Construction and Validation of the Nomogram

**Figure 1:**
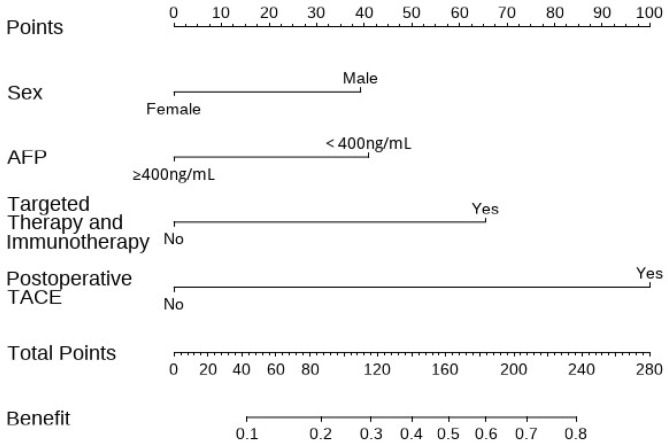
Nomogram to predict the probability of benefiting from the sequential treatment of conversion therapy and radical surgery in HCC patients. HCC, hepatocellular carcinoma; AFP, alpha-fetoprotein; TACE, transcatheter arterial chemoembolization.

**Figure 2:**
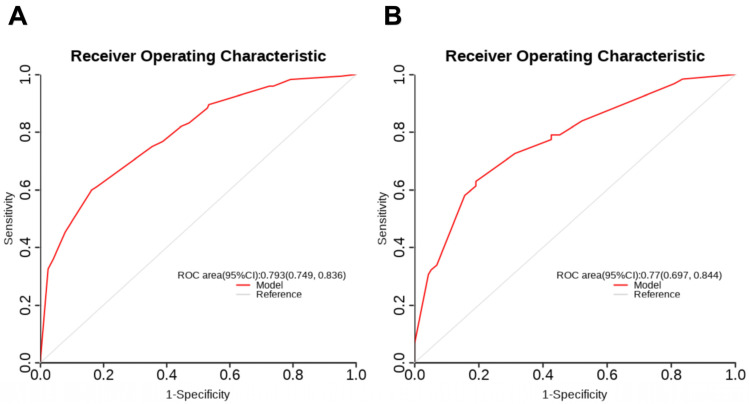
The AUCs of the nomogram for the development (**A**) and validation (**B**) sets. AUC, area under the curve; CI, confidence interval.

As shown in Figure 1, based on the identified independent risk factors in the development set, a nomogram was constructed for this study. As shown in Figure 2, the ROC curves of this nomogram were plotted separately for the development set (Figure 2A) and the validation set (Figure 2B). The AUCs of the nomogram for the development set and the validation set were 0.793 (95% CI: 0.749, 0.836) and 0.770 (95% CI: 0.697, 0.844), respectively. This indicates that the model constructed in this study has good discrimination and performance.

**Figure 3:**
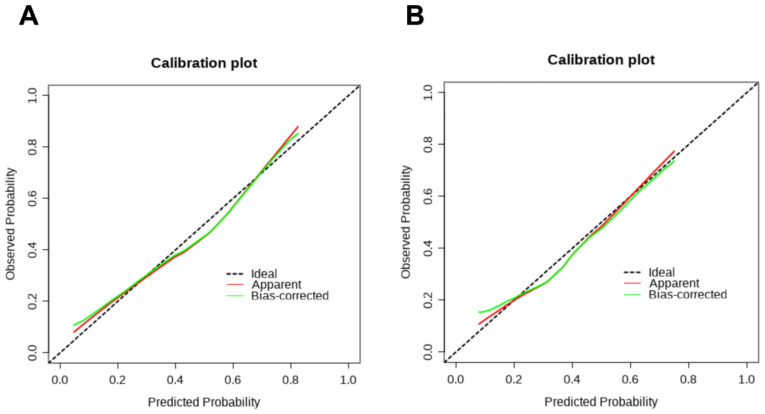
Calibration curves of the nomogram for the development (**A**) and validation (**B**) sets.

As shown in Figure 3, calibration curves of this nomogram were plotted separately for the development set (Figure 3A) and the validation set (Figure 3B) to evaluate its calibration accuracy. The horizontal axis of the calibration curve represents the probability that the model predicts the occurrence of an outcome in HCC patients, while the vertical axis represents the observed probability of the outcome occurring in HCC patients. By analyzing the calibration curves of the development set and the validation set, it can be seen that the probability of an outcome predicted by this model is very close to the observed probability, indicating that the model has good calibration.

**Figure 4:**
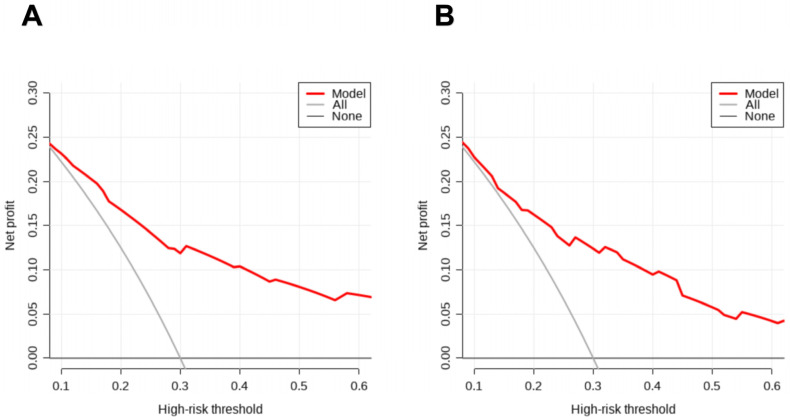
Clinical decision curves of the nomogram for the development (**A**) and validation (**B**) sets.

As shown in Figure 4, the clinical decision curves of the prediction model are plotted for the development set (Figure 4A) and the validation set (Figure 4B). The horizontal axis of the clinical decision curve represents different probability thresholds, and the vertical axis represents the net benefit after subtracting the disadvantages. Through the clinical decision curve, the net benefit of the prediction model under different threshold probabilities in different populations can be quantified to determine the actual clinical application value of the model. In the clinical decision curve of Figure 4, the red line represents the net benefit of using this line graph prediction model for intervention decisions. From this, we can see that using this prediction model to predict the probability of outcomes in HCC patients in clinical practice has good clinical benefits, indicating that the model has ideal clinical practicability.

## 4. Discussion

HCC is one of the most common malignant tumors worldwide. Its onset is insidious, and most patients are diagnosed at an advanced stage, losing the opportunity for radical treatment [10,11]. Transarterial chemoembolization (TACE) has become the standard non-radical treatment option for these advanced-stage patients. However, with the continuous evolution of liver cancer treatment concepts, the role of TACE has expanded beyond palliative care and has become a crucial transformative treatment strategy, demonstrating great potential [12,13].

The core objective of transformative treatment is to effectively reduce the tumor burden and distribution range through initial intervention, thereby converting HCC that was previously deemed “unresectable” or “untransplantable”, due to factors such as tumor size, number, vascular invasion, or insufficient remaining liver volume, to a stage that meets the criteria for radical treatment. In this strategy, TACE, with its unique advantages, is one of the preferred options. In response to this, an increasing number of studies have begun to focus on TACE as a potential transformative treatment option for HCC patients, exploring whether it can offer a curative treatment and even improve the prognosis of patients [14,15].

This study set the two-year overall survival (OS) rate as the outcome after conversion therapy and radical treatment. Logistic regression analysis was used to identify the independent risk factors for this outcome, including sex, AFP, targeted therapy and immunotherapy, and postoperative TACE. The incidence rate of male patients is much higher than that of female patients, and their prognosis is usually worse. This gender difference is not accidental. It is the result of the combined effects of biological behaviors, the hormonal environment, lifestyle, and social–psychological factors. Men have a significantly higher exposure rate to risky behaviors, such as alcohol abuse and smoking, compared with women. These factors not only directly damage liver cells but are also associated with more aggressive tumor biological behaviors [16,17,18].

AFP is not only the most widely used serological tumor marker in the diagnosis of HCC, but also its level is an independent and powerful predictor for evaluating the prognosis of patients. Whether for patients receiving radical treatment or for those receiving palliative treatment, the baseline AFP level before treatment and the dynamic changes after treatment provide crucial information for judging the risk of recurrence and treatment response. The baseline AFP level before treatment directly reflects the biological invasiveness and overall burden of the tumor. Generally, the higher the AFP level is, the poorer the tumor differentiation is and the faster the growth rate is. High AFP levels (typically > 400 ng/mL, especially > 1000 ng/mL) are significantly associated with MVI, incomplete tumor capsule, intrahepatic dissemination, and distant metastasis. These factors directly lead to an increased postoperative recurrence rate and even poor prognosis for HCC patients [19,20,21].

Targeted therapy and immunotherapy have become effective systemic treatment methods that can significantly improve the prognosis of patients with HCC. They exert their effect by inhibiting tumor angiogenesis and the proliferation of tumor cells, as well as activating the patient’s own immune system, to kill the tumors. During the process of conversion therapy, the combination of powerful targeted therapy and immunotherapy with TACE has enabled a significant reduction in and downgrading of a large number of previously inoperable advanced tumors, thereby giving patients the opportunity for radical surgery. Postoperative targeted therapy and immunotherapy as supplementary treatments have further prolonged the survival period of patients. Therefore, targeted therapy and immunotherapy are important components in the process by which HCC patients benefit from radical treatment after undergoing transformation therapy [22,23,24].

The high recurrence rate after radical treatment of HCC is the main obstacle affecting the long-term survival of patients. TACE aims to eliminate residual tiny lesions and prevent tiny metastasis after surgery, thereby reducing the risk of recurrence and improving prognosis. For patients who have undergone radical treatment and subsequent conversion therapy, it usually means that they had a relatively severe tumor burden before the surgery. For HCC patients with high-risk recurrence factors, postoperative adjuvant TACE can significantly reduce the tumor recurrence rate, prolong the recurrence-free survival period, and possibly bring benefits to the overall survival [25,26,27].

Based on the characteristics of HCC patients who benefited from the sequential treatment of conversion therapy and radical surgery, we constructed a nomogram to predict whether HCC patients could benefit from the sequential treatment of conversion therapy and radical surgery. The evaluation through the AUC, calibration curve, and clinical decision curve indicates that this nomogram has good predictive performance. Through this model, we can preliminarily determine before the intervention whether HCC patients can benefit from the sequential treatment of conversion therapy and radical surgery and also determine some treatment strategies in advance to improve the prognosis of the patients. For instance, in the case of HCC patients with a high baseline AFP level, before implementing the intervention, we need to reduce the AFP level. This may be achieved through multiple local TACE treatments. Furthermore, for HCC patients who have undergone conversion therapy, if their financial situation permits, targeted therapy and immunotherapy should be combined with TACE to reduce the tumor burden of the patients. This may help patients receive subsequent radical treatment and benefit from it. After patients undergo conversion therapy and radical treatment, for HCC patients with high-risk recurrence factors, some postoperative adjuvant treatments should be adopted, such as TACE, targeted therapy, and immunotherapy. These might be the key factors that can prolong the survival of patients. Therefore, the nomogram constructed in this study has certain practicality in clinical applications and also demonstrates good predictive performance. The variables used in it are also easy to obtain and there are no restrictions on their usage.

However, this study also has certain limitations. Firstly, this study is a single-center study and lacks multi-center data to support the conclusions of this research. Moreover, this study is a retrospective study and requires further prospective studies to prove its feasibility. Secondly, the nomogram constructed in this study is only applicable to predicting whether HCC patients can benefit from the sequential treatment of conversion therapy and radical surgery. Furthermore, some of the postoperative variables included in this study further limit the application scope of the model. And its ability to predict the prognosis of other HCC patients after intervention has not yet been proven.

## 5. Conclusions

This study developed a nomogram for predicting whether HCC patients can benefit from the sequential treatment of conversion therapy and radical surgery. This is of great help for us to formulate treatment strategies for patients with advanced HCC.

## Figures and Tables

**Table 1:** Clinical characteristics of development set and validation set.

Variables		Development Set (*n* = 412)	Validation Set (*n* = 177)	*p* Value
Sex	Male	342 (83.0%)	153 (86.4%)	0.297
	Female	70 (17.0%)	24 (13.6%)	
Age		54 ± 11	53 ± 11	0.329
BCLC stage	A	128 (31.1%)	54 (30.5%)	0.893
	B/C	284 (68.9%)	123 (69.5%)	
Child–Pugh score	A	407 (98.8%)	174 (98.3%)	0.644
	B	5 (1.2%)	3 (1.7%)	
Tumor number	Single	127 (30.8%)	60 (33.9%)	0.463
	Multiple	285 (69.2%)	117 (66.1%)	
Max. tumor size (mm)		62 (40–99)	59 (41–89)	0.895
AFP ( ≥ 400ng/mL)	No	276 (67.0%)	112 (63.3%)	0.383
	Yes	136 (33.0%)	65 (36.7%)	
ALT (U/L)		38 (26–60)	38 (26–59)	0.471
AST (U/L)		44 (31–71)	42 (30–72)	0.635
TBIL (umol/L)		13.2 (9.5–19.1)	12.3 (8.8–18.6)	0.105
PNI		46.7 (43.5–50.6)	46.9 (44.3–50.9)	0.465
Preoperative TACE number		3 (2–5)	3 (2–4)	0.498
Tumor differentiation	Poor/Moderate	281 (68.2%)	115 (65.0%)	0.444
	Good	131 (31.8%)	62 (35.0%)	
MVI	0	296 (71.8%)	117 (66.1%)	0.270
	1	100 (24.3%)	49 (27.7%)	
	2	16 (3.9%)	11 (6.2%)	
Targeted therapy and immunotherapy	No	209 (50.7%)	106 (59.9%)	0.041
	Yes	203 (49.3%)	71 (40.1%)	
Postoperative TACE	No	227 (55.1%)	103 (58.2%)	0.488
	Yes	185 (44.9%)	74 (41.8%)	
Benefit	No	240 (58.3%)	115 (65.0%)	0.127
	Yes	172 (41.7%)	62 (35.0%)	
Status	Alive	57 (13.8%)	37 (20.9%)	0.032
	Dead	355 (86.2%)	140 (79.1%)	
OS (months)		22.0 (15.0–36.0)	19 (14.0–32.5)	0.113

BCLC, Barcelona Clinic Liver Cancer; AFP, alpha-fetoprotein; ALT, alanine aminotransferase; AST, aspartate transferase; TBIL, total bilirubin; PNI, prognostic nutritional index; TACE, transcatheter arterial chemoembolization; MVI, microvascular invasion; OS, overall survival.

**Table 2:** Univariable and multivariable Logistic regression analysis of benefitting from radical treatment after conversion therapy in development set.

Variables	Univariable Analysis		Multivariable Analysis	
	OR (95% CI)	*p* Value	OR (95% CI)	*p* Value
Sex (Male)	2.000 (1.142, 3.504)	0.015	2.000 (1.018, 3.931)	0.044
Age	0.996 (0.979, 1.013)	0.631		
BCLC stage (B/C)	0.449 (0.287, 0.702)	<0.001	0.666 (0.298, 1.488)	0.321
Child–Pugh score (B)	0.345 (0.038, 3.114)	0.343		
Tumor number (multiple)	0.589 (0.381, 0.912)	0.018	1.154 (0.525, 2.538)	0.722
Max. tumor size	0.986 (0.980, 0.992)	<0.001	0.994 (0.986, 1.002)	0.130
AFP ( ≥ 400ng/mL)	0.478 (0.309, 0.739)	0.001	0.533 (0.313, 0.907)	0.020
ALT	0.995 (0.991, 0.999)	0.013	0.999 (0.990, 1.008)	0.811
AST	0.993 (0.988, 0.997)	0.002	0.995 (0.985, 1.004)	0.247
TBIL	1.001 (0.998, 1.004)	0.463		
PNI	1.041 (1.004, 1.080)	0.032	1.029 (0.985, 1.076)	0.197
Preoperative TACE number	0.883 (0.787, 0.991)	0.035	1.032 (0.882, 1.208)	0.692
Tumor differentiation (good)	0.969 (0.636, 1.476)	0.882		
MVI	1.178 (0.824, 1.685)	0.369		
Targeted therapy and immunotherapy (yes)	3.485 (2.308, 5.261)	<0.001	3.283 (2.029, 5.312)	<0.001
Postoperative TACE (yes)	5.919 (3.850, 9.101)	<0.001	6.544 (4.021, 10.649)	<0.001

OR, odds ratio; CI, confidence interval; BCLC, Barcelona Clinic Liver Cancer; AFP, alpha-fetoprotein; ALT, alanine aminotransferase; AST, aspartate transferase; TBIL, total bilirubin; PNI, prognostic nutritional index; TACE, transcatheter arterial chemoembolization; MVI, microvascular invasion.

## Data Availability

The datasets generated during and/or analyzed during the current study are available from the corresponding author.
